# Expression levels and clinical significance of ferroptosis-related genes in patients with myocardial infarction

**DOI:** 10.1038/s41598-023-49336-2

**Published:** 2024-01-22

**Authors:** Lei Huang, Xiaoyang Wang, Bin Hu, Shuling Rong

**Affiliations:** Present Address: Second Hospital of Shanxi Medical University, Shanxi Medical University, Taiyuan, 030000 China

**Keywords:** Cardiology, Gene regulation

## Abstract

Myocardial infarction (MI) is the most serious type of cardiovascular disease and the leading cause of cardiac death.Ferroptosis is one of the newly discovered programmed cell death modes in MI, but its mechanism of action in MI has not been clarified.In this study, we analyzed the expression changes of ferroptosis-related genes in MI and explored the potential mechanisms of ferroptosis-related functions in myocardial infarction. Public data sets GSE19339, GSE97320 and GSE141512 were retrieved from the Gene Expression Omnibus (GEO) Datasets public database. After data preprocessing, differentially expressed genes were screened, and differentially expressed ferroptosis-related genes associated with myocardial infarction were obtained. The biological function and signaling pathway enrichment analysis were performed to establish the PPI interaction network specific to heart tissue, and the differential diagnosis significance of differentially expressed ferroptosis-related genes associated with myocardial infarction was analyzed by ROC curve and decision tree model.A total of 317 genes showed significant changes in expression levels in patients with myocardial infarction, including 205 down-regulated genes and 112 up-regulated genes.Gene Ontology (GO) enrichment analysis and functional classification of Kyoto Encyclopedia of Genes and Genomes (KEGG) signaling pathways showed that these genes were mainly involved in signaling pathways or biological functions related to inflammation and apoptosis.Five differentially expressed ferroptosis-related genes (SLC2A3, EPAS1, HMOX1, ATM, FANCD2) were obtained, all of which played key biological functions in cardiac tissue function. SLC2A3, EPAS1, HMOX1, ATM and FANCD2 genes all had good diagnostic value for myocardial infarction (P < 0.05). The increase of SLC2A3, EPAS1 and HMOX1 are risk factors for myocardial infarction, while ATM and FANCD2 are protective factors.Decision tree analysis showed that SLC2A3, HMOX1, ATM, FANCD2 gene had higher net yield in diagnosing myocardial infarction. In summary, the mechanism of ferroptosis is involved in the occurrence and progression of myocardial infarction. In this study, five differentially expressed ferroptosis-related genes associated with myocardial infarction were retrieved, which may be good biomarkers of ferroptosis after MI.These findings also suggest that the differential expression of ferroptosis-related genes associated with myocardial infarction has significant diagnostic significance for myocardial infarction.

Myocardial infarction (MI) is the most serious type of cardiovascular disease and the leading cause of cardiac death. Ferroptosis is one of the newly discovered programmed cell death modes in MI, but its mechanism of action in MI has not been clarified. In this study, we analyzed the expression changes of ferroptosis-related genes in MI and explored the potential mechanisms of ferroptosis-related functions in myocardial infarction. Public data sets GSE19339, GSE97320 and GSE141512 were retrieved from the Gene Expression Omnibus (GEO) Datasets public database. After data preprocessing, differentially expressed genes were screened, and differentially expressed ferroptosis-related genes associated with myocardial infarction were obtained. The biological function and signaling pathway enrichment analysis were performed to establish the PPI interaction network specific to heart tissue, and the differential diagnosis significance of differentially expressed ferroptosis-related genes associated with myocardial infarction was analyzed by ROC curve and decision tree model. A total of 317 genes showed significant changes in expression levels in patients with myocardial infarction, including 205 down-regulated genes and 112 up-regulated genes. Gene Ontology (GO) enrichment analysis and functional classification of Kyoto Encyclopedia of Genes and Genomes (KEGG) signaling pathways showed that these genes were mainly involved in signaling pathways or biological functions related to inflammation and apoptosis. Five differentially expressed ferroptosis-related genes (SLC2A3, EPAS1, HMOX1, ATM, FANCD2) were obtained, all of which played key biological functions in cardiac tissue function. SLC2A3, EPAS1, HMOX1, ATM and FANCD2 genes all had good diagnostic value for myocardial infarction (*P* < 0.05). The increase of SLC2A3, EPAS1 and HMOX1 are risk factors for myocardial infarction, while ATM and FANCD2 are protective factors.Decision tree analysis showed that SLC2A3, HMOX1, ATM, FANCD2 gene had higher net yield in diagnosing myocardial infarction. In summary, the mechanism of ferroptosis is involved in the occurrence and progression of myocardial infarction. In this study, five differentially expressed ferroptosis-related genes associated with myocardial infarction were retrieved, which may be good biomarkers of ferroptosis after MI. These findings also suggest that the differential expression of ferroptosis-related genes associated with myocardial infarction has significant diagnostic significance for myocardial infarction.

With the aging of the population and the acceleration of urbanization, cardiovascular diseases gradually pose a serious threat to human life and health, and are the main cause of death worldwide. The incidence and mortality of cardiovascular diseases are still on the rise in China. Myocardial infarction (MI) is an important cause of death^1–3^. MI is caused by severe persistent ischemia and hypoxia of some myocardium tissue caused by coronary artery occlusion, resulting in local or extensive myocardial cell damage and necrosis, and in severe cases, arrhythmia, cardiogenic shock or heart failure may occur, or even endanger the life of patients^2,4^. Although reperfusion and revascularization therapies such as thrombolysis and percutaneous coronary intervention have successfully reduced the scope of MI and improved clinical prognosis^5,6^, the pathogenesis still remains unclear.

The occurrence of MI is often accompanied by damage to the heart structure and function, and irreversible cardiomyocyte death can occur.However,cardiomyocyte death is considered to be the initiation and main event of MI. The cell death that occurs in either a passive way or an actively mediated cell suicide program is collectively known as programmed cell death (PCD)^7^. Various forms of cellular PCD play an important role in the occurrence and development of MI, among which ferroptosis is one of the new types of PCD that has attracted attention in recent years^8,9^. Baba et al.^10^ confirmed for the first time that ferroptosis existed in MI cardiomyocytes, and the use of rapamycin could inhibit ferroptosis in cardiomyocytes by reducing the production of ROS. Park et al.^11^ confirmed that ferroptosis of cardiomyocytes can be induced by down-regulation of GPX4 during MI. Tang et al.^12^ found that the expression of miR-30d in cardiomyocytes was reduced after MI, which inhibited autophagy of cardiomyocytes and on the contrary promoted ferroptosis of cardiomyocytes. Song et al.^13^ also confirmed that exosomes from bone marrow mesenchymal stem cells in human cord blood can inhibit the expression of DMT1 in cardiomyocytes through miR-23a-3p, thereby inhibiting ferroptosis in cardiomyocytes. However, the pathogenesis of ferroptosis and MI remains to be further explored. Fang et al. found that the iron content in cardiomyocytes of mice lacking ferritin decreased, while the oxidative stress level increased. Overexpression of iron regulatory gene SLC7A11 could inhibit ferroptosis and reduce myocardial cell damage^3^. It can be seen that ferroptosis-related genes may become new biomarkers or therapeutic targets for cardiovascular diseases, so more studies are needed to explore ferroptosis related genes.

Based on bioinformatics methods and data sets of public databases, this study analyzed the changes in the expression level of ferroptosis-related genes after myocardial infarction, its main biological functions and signaling pathways involved, as well as the clinical value for the differential diagnosis of patients with myocardial infarction, aiming to explore the influence of ferroptosis-related mechanisms on myocardial infarction and its clinical significance.

## Materials and methods

### Retrieval and acquisition of data sets

Firstly, data sets of “Patients with Myocardial infarction” VS “Healthy controls” were screened in the GEO Datasets public database (https://www.ncbi.nlm.nih.gov/GEO/) with the keyword “Myocardial infarction” and finally included and downloaded from GSE19339 (https://www.ncbi.nlm.nih.gov/geo/query/acc.cgi?acc=GSE19339), GSE97320 (https://www.ncbi.nlm.nih.gov/geo/query/acc.cgi), GSE141512 (https://www.ncbi.nlm.nih.gov/geo/query/acc.cgi). All data sets were Case–control study designed, and the subjects were divided into two groups: Case Group (N = 11) and control group (N = 11).All data were Array Chip data.

### Ferroptosis-related genes

In this study,using a well-documented list of human ferroptosis-related genes, 259 ferroptosis-related genes were from the FerrDb V2 database (http://www.zhounan.org/ferrdb/current/).

### Data preprocessing

The difference between batches of multiple data sets was eliminated by using Network Analys (https://www.networkanalyst.ca) online software to improve the stability of the original data. Then the original chip data is standardized (Log_2_Counts per million) so that the data can be combined for analysis.

### Differential expression genes screening

After data preprocessing, the original chip data were analyzed by R language Limma bag, and the differentially expressed genes related to myocardial infarction were screened with the following screening criteria: adjusted *P* < 0.05 and log2(Fold Change) > 1 or log2(Fold Change) < − 1. The differentially expressed genes related to myocardial infarction (MI) and ferroptosis-related genes were obtained by crossing the lists of MI-related differentially expressed genes and human ferroptosis-related genes.

### Enrichment analysis

In order to understand in detail the biological functions of differentially expressed ferroptosis-related genes associated with myocardial infarction and the signaling pathways involved, GO enrichment analysis and KEGG^14–16^ signaling pathway enrichment analysis (https://www.kegg.jp/kegg/kegg1.html) were performed by using online software STRING (https://string-db.org/). The former includes biological process (BP), molecular function (MF) and cellular components (CC), and then is visualized and mapped.The main parameters of enrichment analysis:* P* < 0.05 or FDR < 0.05 were considered to have statistical significance—the scale on the right of the enrichment diagram − Log10 (Adj.*P*) > 1.3; Different colors in the figure represented the significance of differential enrichment results, and the larger value indicated the smaller FDR value. The size of the circle represented the number of enriched genes, and the larger circle indicated the more enriched genes.

### Establishment of cardiac tissue specific PPI network

The PPI network of differentially expressed ferroptosis-related genes related to myocardial infarction was established by using STRING database. The parameter was set to Confidence = 0.7, and the final PPI network was established. Finally, Cytoscape was adopted for visualization, and the PPI network map was obtained.

### Establishment of decision tree model

The list of differentially expressed genes related to ferroptosis associated with myocardial infarction was taken as the independent variable, and the sample sizes of the data sets GSE19339, GSE97320, and GSE141512 were randomly divided into test sets and verification sets according to the system, and decision curve analysis (DCA) was conducted. A decision tree model was established to investigate the clinical indicator value of differentially expressed ferroptosis-related genes in myocardial infarction.

### Establishment of ROC curve

Receiver operating characteristic curve (ROC) was used to screen the diagnostic value of differentially expressed ferroptosis-related genes associated with myocardial infarction, and to evaluate their potential clinical value as biomarkers associated with myocardial infarction.

### Statistical analysis

SPSS 26.0 statistical software was used, with myocardial infarction as the result variable, and ROC curve analysis was used to screen the influencing factors of ferroptosis-related genes on the diagnosis of myocardial infarction. *P* < 0.05 was considered statistically significant.

## Results

### Data processing and differential expression screening

**Figure 1:**
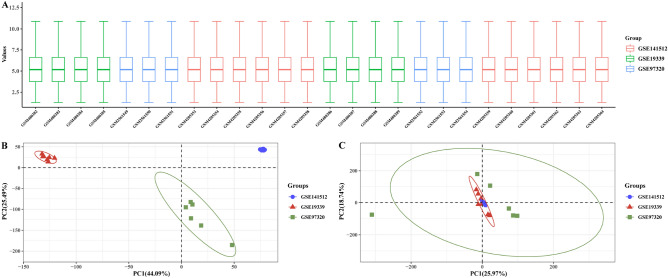
Results of data processing.

After the original data of the three data sets were standardized, a box-like diagram was drawn. As can be seen from the diagram, the mean distribution of the gene expression data of each sample was basically located in the center line of the box and in a straight line, indicating that the difference between batches was eliminated effectively and the data was stable (Fig. 1A–C).

**Figure 2:**
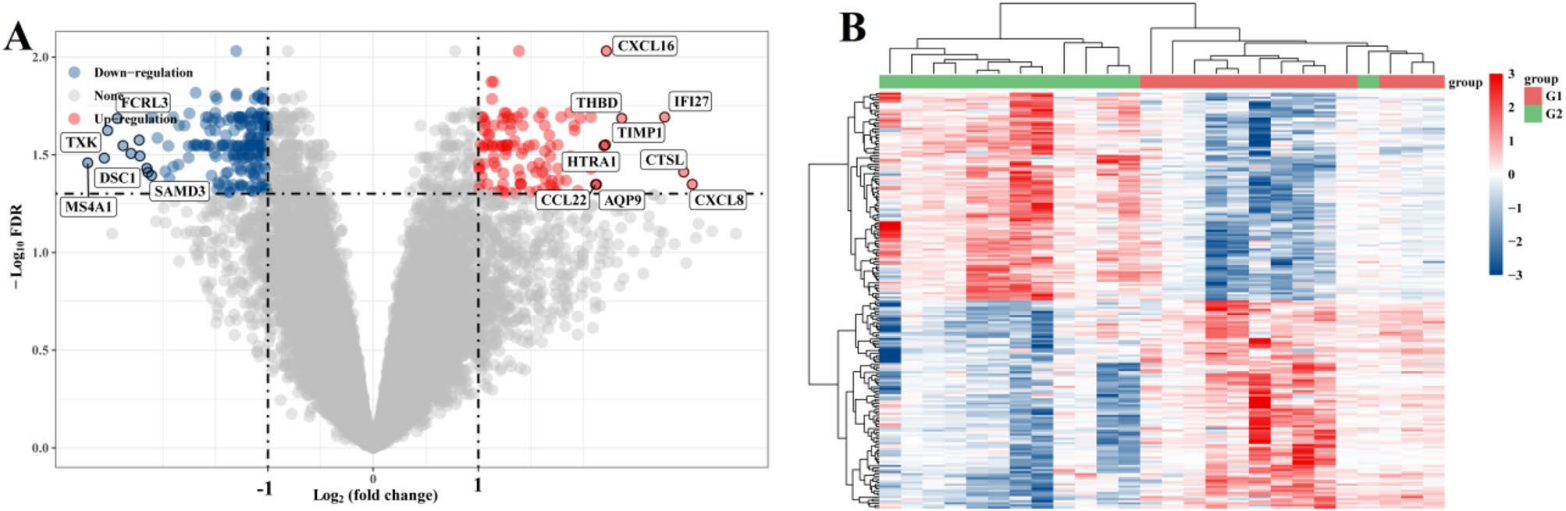
(**A**) Volcano map of differentially expressed genes after screening (adjusted *P* < 0.05 and log2(Fold Change) > 1 or log2(Fold Change) < − 1). (**B**) cluster heat map.

Compared with the control group, the expression levels of 317 genes in the myocardial infarction patients group were significantly changed, including 205 down-regulated genes and 112 up-regulated genes, and the volcano map and cluster heat map of differentially expressed genes (Fig. 2A–B).

### Enrichment analysis

**Figure 3:**
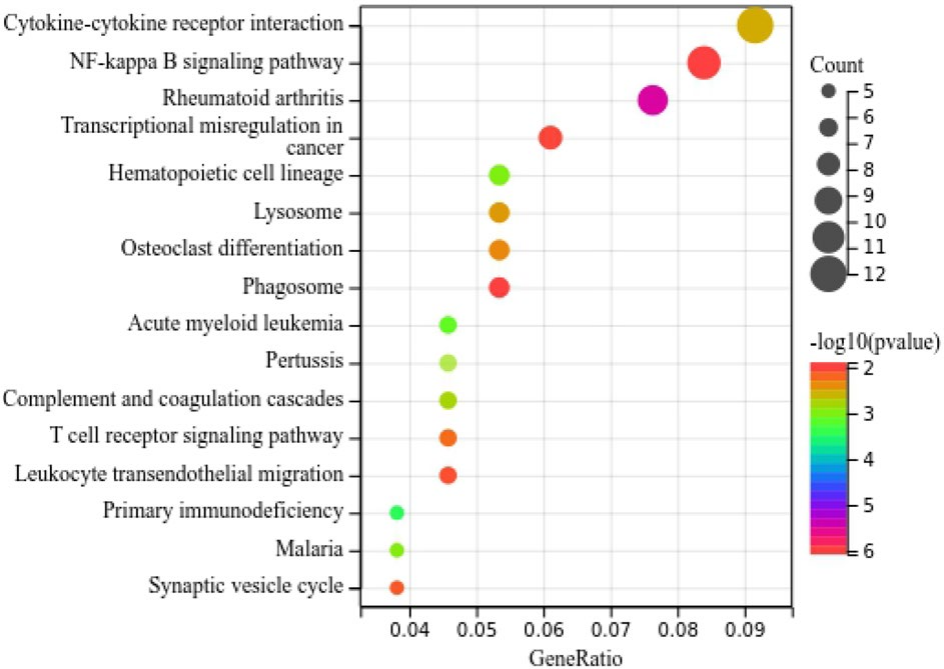
Enrichment analysis of differentially expressed genes.

GO and KEGG enrichment analysis showed that: Differentially expressed genes related to myocardial infarction were mainly involved in Cytokine–cytokine receptor interaction, NF-kappa B signaling pathway and other signaling pathways or biological functions, and played a key role (Fig. 3).

### Screening of genes related to differential ferroptosis

**Figure 4:**
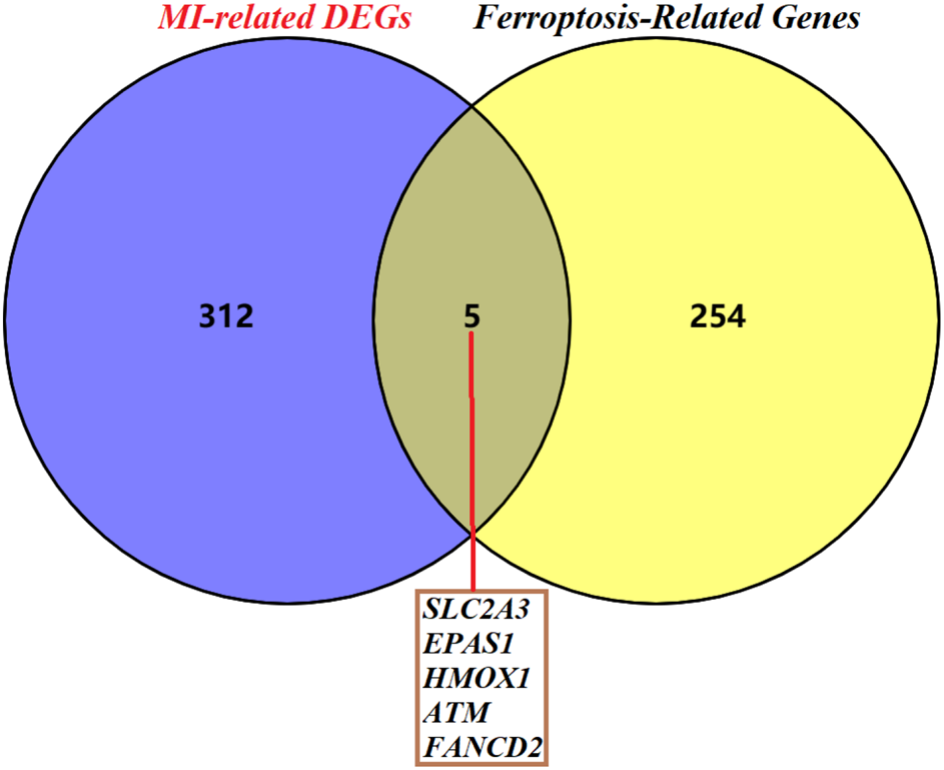
Screening of differentially expressed ferroptosis genes associated with myocardial infarction.

The intersection of 317 differentially expressed genes and the list of human ferroptosis-related genes showed that 5 differentially expressed ferroptosis-related genes were obtained, including SLC2A3, EPAS1, HMOX1, ATM, and FANCD2, as shown in Fig. 4.

### Establishment of cardiac tissue specific PPI network

**Figure 5:**
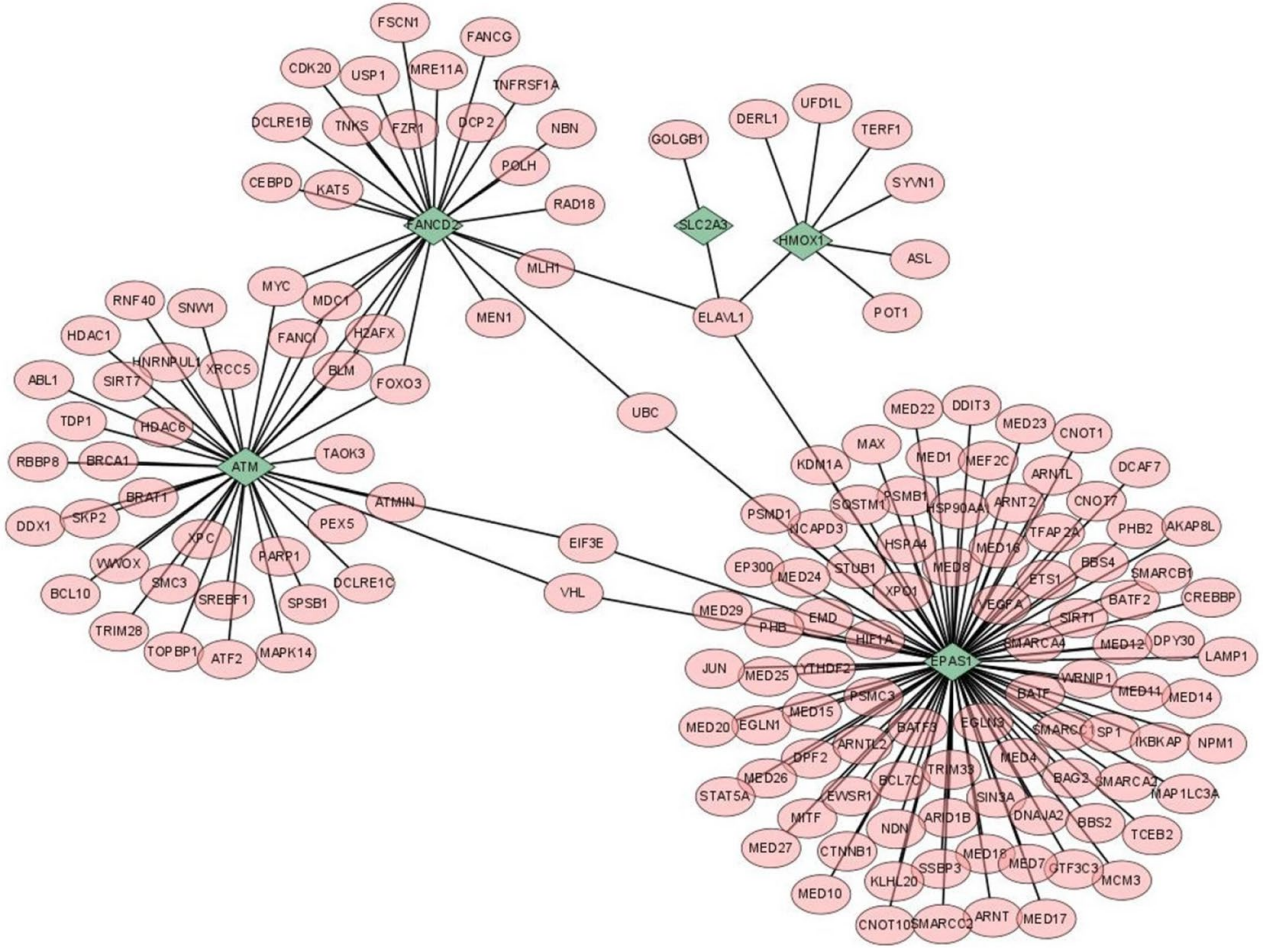
Establishment of PPI network with differentially expressed ferroptosis gene associated with myocardial infarction.

The PPI networks of 5 MI related differentially expressed ferroptosis gene decoder proteins are shown in Fig. 5. It can be seen from the figure that 5 MI related differentially expressed ferroptosis proteins all played important biological functions in the function of heart tissue, suggesting that the mechanism related to ferroptosis may play an important role in the occurrence and development of myocardial infarction. And it was realized through SLC2A3, EPAS1, HMOX1, ATM and FANCD2 discovered in this study.

### Establishment of ROC curve

**Figure 6:**
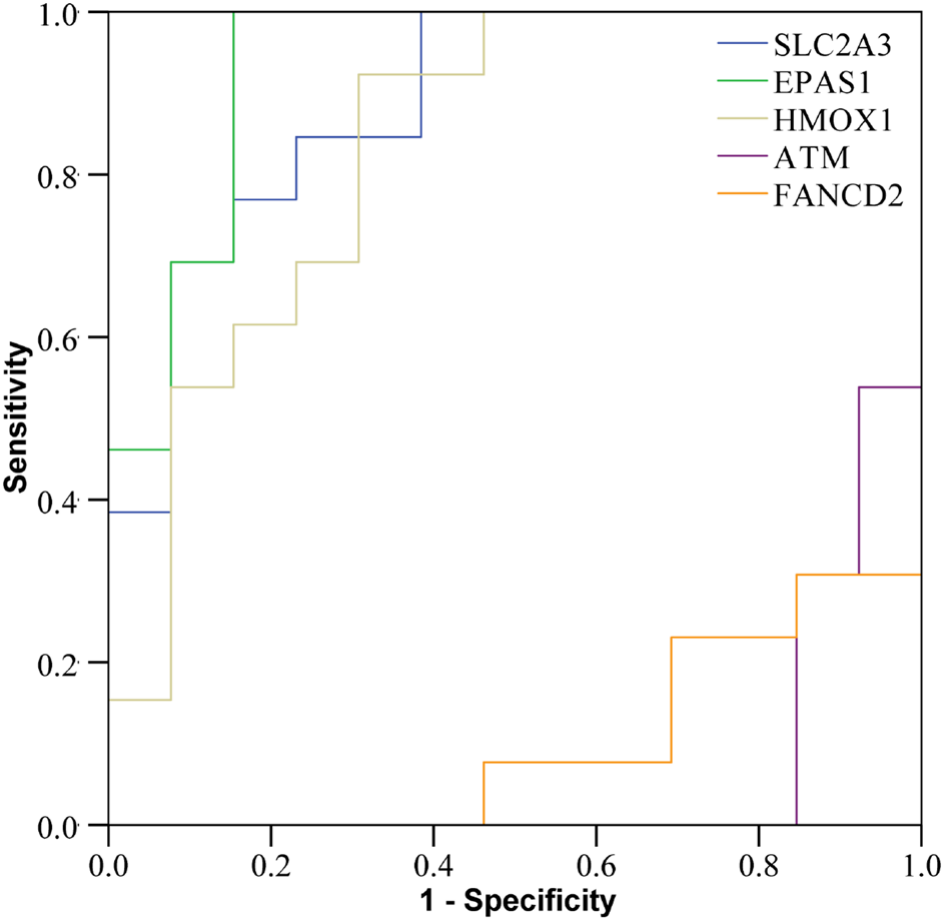
ROC curve of genes associated with myocardial infarction of related differential ferroptosis.

ROC curves of 5 differentially ferroptosis-related genes associated with myocardial infarction showed that SLC2A3, EPAS1, HMOX1, ATM and FANCD2 genes had good diagnostic value for myocardial infarction, and the area under the curve had statistical significance (*P* < 0.05). The increase of SLC2A3, EPAS1 and HMOX1 were risk factors for myocardial infarction, while ATM and FANCD2 were protective factors(Fig. 6).

### Establishment of decision tree model

**Figure 7:**
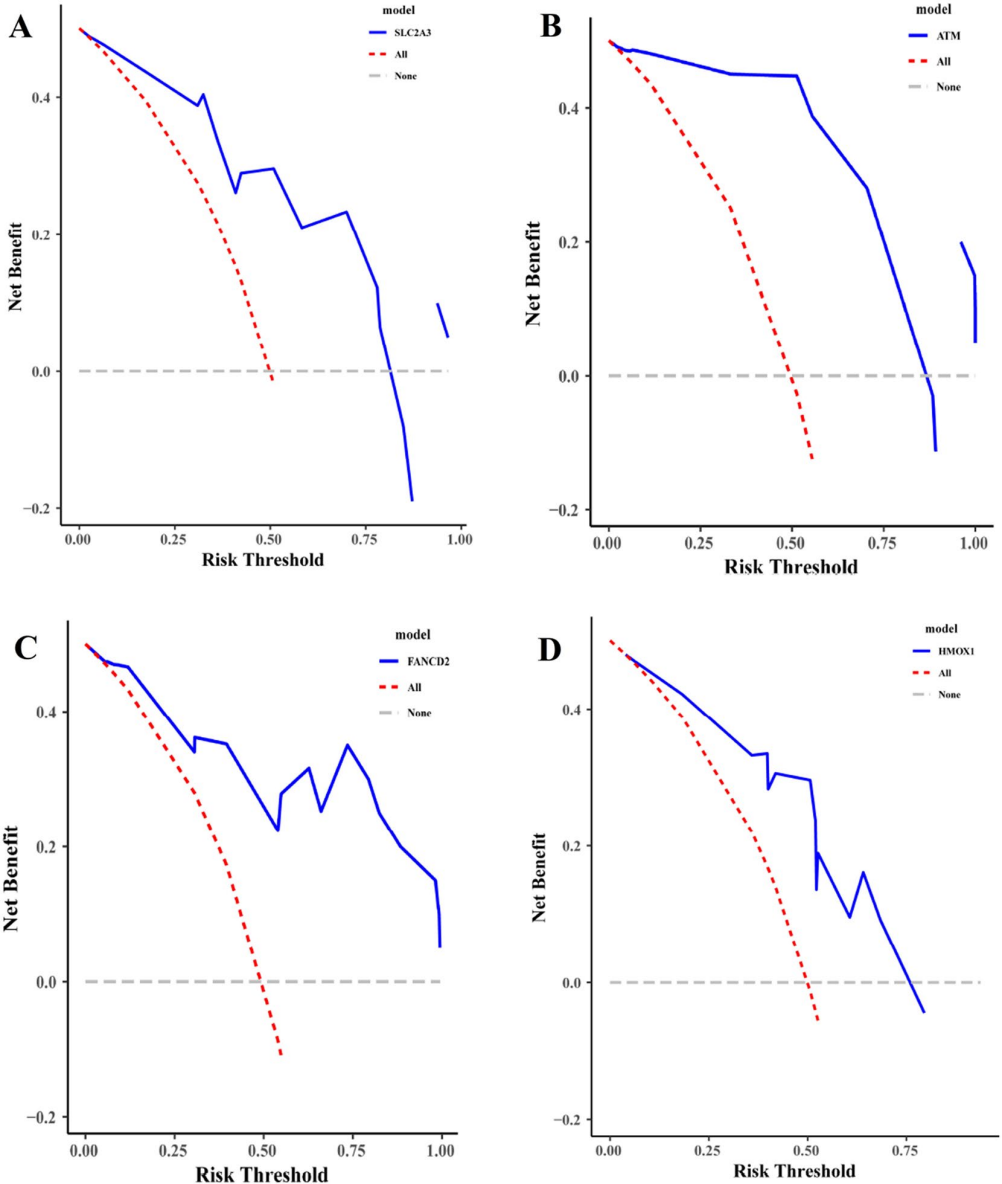
ROC curve of myocardial infarction related differences in ferroptosis-related genes (training set).

**Figure 8:**
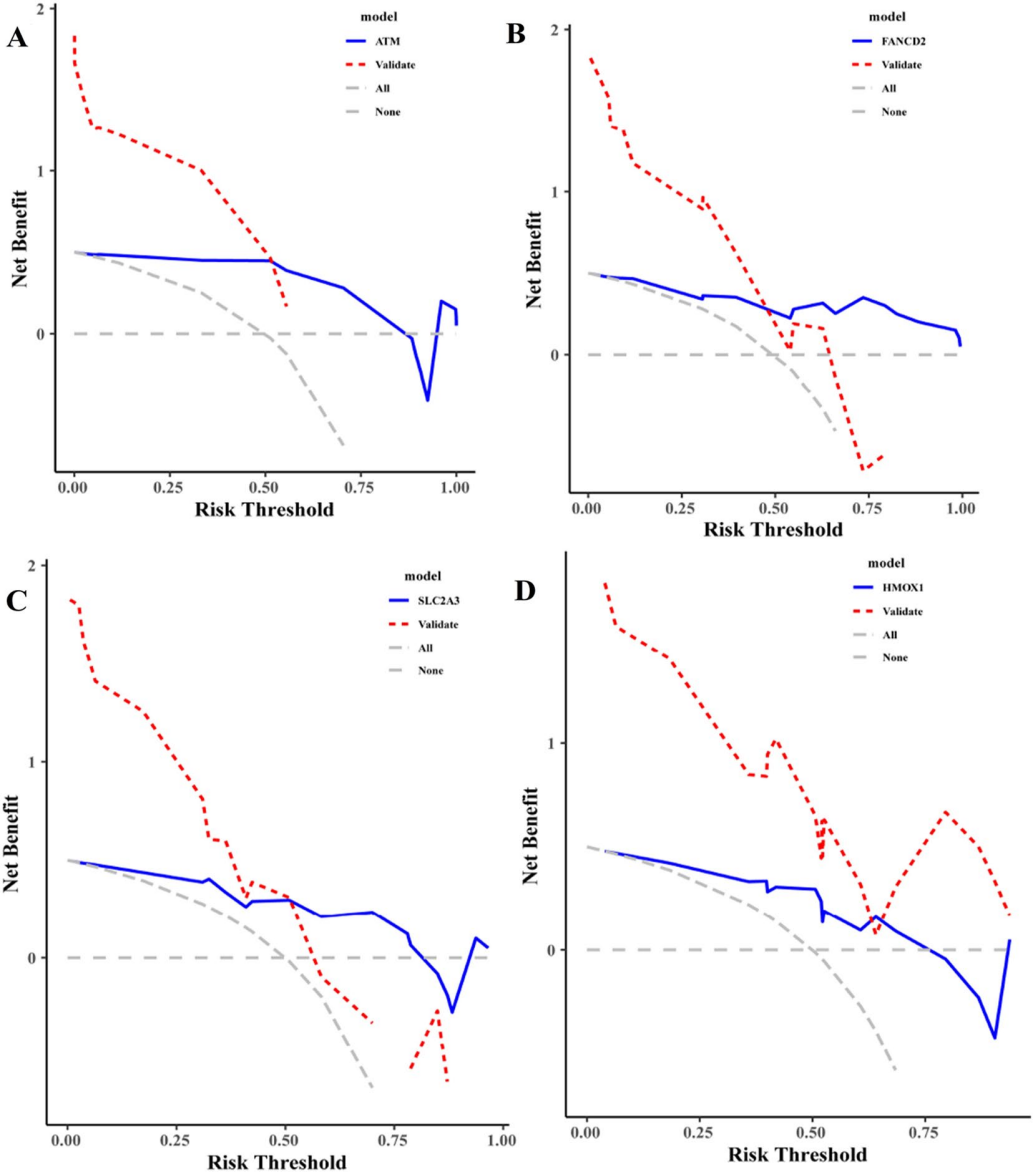
ROC curve of myocardial infarction related differences in ferroptosis-related genes (validation set).

According to the method of systematic random sampling, the sample size was divided into test set and verification set, and the decision tree model of SLC2A3, EPAS1, HMOX1, ATM and FANCD2 genes on myocardial infarction was established. The results showed that SLC2A3, HMOX1, ATM and FANCD2 genes had higher net yield in the diagnosis of myocardial infarction. In addition, the results of test set and verification set were basically the same, as shown in Figs. 7, 8.

## Discussion

Acute myocardial infarction has become a serious public health problem in China and even in the world. Myocardial infarction is considered to be an acute coronary syndrome that seriously harms human health based on the interaction between individual genes and living environment, and is a serious adverse cardiovascular event. Despite the development of half a century and some breakthroughs in diagnosis and treatment methods, there are still major limitations, and cardiomyocytes with poor regenerative potential cannot be effectively repaired. After myocardial infarction, a variety of factors lead to the death of cardiomyocytes, and excessive death of cardiomyocytes will lead to a sudden decrease in the number of cardiomyocytes, which will lead to defects in the structure and function of the heart, and accelerate the occurrence of heart failure^17,18^. The balance between proliferation and death of cardiomyocytes is an important pathological basis to reduce the risk of myocardial infarction, and is also a key strategy to protect heart function and prevent heart disease. At present, it is known that multiple cell death modes have been discovered^19–21^. The potential role of ferroptosis as a novel cell death mode in myocardial infarction has been confirmed. Therefore, the active study of ferroptosis-related genes is helpful to elucidate the pathogenesis of MI.

In this study, biological information was used to identify key genes related to ferroptosis in patients with myocardial infarction. The GSE dataset downloaded from GEO Datasets public database and intersected with FerrDb v2 database revealed significant changes in the expression of 5 genes related to ferroptosis, including: SLC2A3, EPAS1, HMOX1, ATM, FANCD2. Enrichment analysis showed that these genes were mainly involved in the biological functions and signaling pathways related to inflammatory response and apoptosis, so it was speculated that the mechanism of ferroptosis might be involved in the occurrence and progression of myocardial infarction by regulating inflammatory response and apoptosis. ROC curves of 5 different genes related to ferroptosis associated with myocardial infarction showed that SLC2A3, EPAS1, HMOX1, ATM and FANCD2 genes had good diagnostic value for myocardial infarction, and the elevation of SLC2A3, EPAS1 and HMOX1 was a risk factor for myocardial infarction. ATM and FANCD2 were protective factors.

Some researchers used gene sequencing technology to explore the key regulatory factors of ferroptosis mechanism in adriamycin cardiomyopathy models. Similar to the results of this study, they also found that the expression level of heme oxygenase-1 (Hmox1) gene was significantly increased. The cardiac function of doxorubicin mice was obviously protected after Hmox1 inhibitor, but the effect was opposite after Hmox1 overexpression. Subsequent experiments confirmed that the activation of Hmox1 mediates the release of free iron ions from heme and their subsequent accumulation in cardiomyocytes, thus inducing the occurrence of ferroptosis^22^. Previous studies revealed that SLC2A3 played a key role in macrophage infiltration and transformation. The main infiltrating immune cells in myocardial infarction were macrophages, which coordinated the inflammatory response by differentiating into different pro-inflammatory or pro-healing subgroups, promoted the clearance of damaged tissues, and played an active role in left ventricular remodeling^23^. On the other hand, excessive inflammatory response would increase the size of myocardial infarction and affect wound repair, resulting in long-term heart failure^24^. Based on the results of this study and previous experimental evidence, ferroptosis and inflammation played a key role in the occurrence of myocardial infarction, affecting the heart function and prognosis of patients, and even had important significance for patients' quality of life.However, due to the small total number of samples in the dataset included in this study, this study is a preliminary study, and the results obtained need to be further verified by more rigorous independent experiments in future studies.

In conclusion, the expression of ferroptosis-related genes changed significantly after the occurrence of myocardial infarction, suggesting that the mechanism of ferroptosis was involved in the occurrence and progression of myocardial infarction. The differentially expressed genes related to ferroptosis associated with myocardial infarction were mainly involved in signaling pathways or biological functions related to inflammation and apoptosis. The differentially expressed ferroptosis-related genes associated with myocardial infarction have significant diagnostic and differential significance for myocardial infarction.

## Data Availability

The gene expression profiles of GSE19339,GSE97320 and GSE141512 were downloaded from Gene Expression Omnibus (GEO) (https://www.ncbi.nlm.nih.gov/geo/query/acc.cgi?acc=GSE19339,https://www.ncbi.nlm.nih.gov/geo/query/acc.cgi?acc=GSE97320 and https://www.ncbi.nlm.nih.gov/geo/query/acc.cgi?acc=GSE141512).
